# Implementation of a context-specific accreditation assessment tool for affirming quality midwifery education in Bangladesh: a qualitative research study

**DOI:** 10.1080/16549716.2020.1761642

**Published:** 2020-05-20

**Authors:** Malin Bogren, Afroza Banu, Shahanaj Parvin, Merry Chowdhury, Kerstin Erlandsson

**Affiliations:** a Institute of Health and Care Sciences, Sahlgrenska Academy, University of Gothenburg, Gothenburg, Sweden; b Directorate General of Nursing and Midwifery, Sher-E-Bangla Nagar Nursing College, Dhaka, Bangladesh; c Chattogram Nursing College, Chattogram Nursing College, Chattogram, Bangladesh; d School of Education, Health and Social Studies, Dalarna University, Falun, Sweden

**Keywords:** Accreditation/standards, midwifery/education, quality improvement, South Asia, Bangladesh

## Abstract

**Background**: Only recently did midwifery become a profession in Bangladesh. As such, sufficient quality education, both theory and practice, remains a challenge. In 2018, a context-specific accreditation assessment tool for affirming quality midwifery education was therefore developed and implemented.

**Objectives**: To describe both the positive and negative aspects of the implementation of an accreditation process at midwifery education institutions in Bangladesh and to sketch out areas for possible improvement.

**Method**: Forty focus group discussions were conducted with 276 policymakers, regulatory authorities and educators involved in midwifery education and services in Bangladesh. The Consolidated Framework for Implementation Research (CFIR) was used in a directed content analysis approach.

**Results**: The accreditation assessment tool was developed using a participatory and consensus-building approach, building on existing policies, which resulted in the national ownership of its implementation. Staff from clinical sites were not included in the accreditation process; unless this changes, this will make it difficult for Bangladesh to achieve the set accreditation standards. The accreditation process has improved communication between the midwifery teaching institutions, policymakers and regulatory authorities. Educators started to visit the clinical sites more frequently. The planning process was complex and time-consuming, and emphasis was put on the importance of developing a plan of action for measuring improvements.

**Conclusion**: In the move from the initial assessment of an accreditation process to its implementation, it is essential to make public the results found at all educational institutions. This encourages acceptance, while soliciting feedback and suggestions for future action. Only then can an accreditation process have an impact on the provision of high-quality midwifery education and services. This paper aims to encourage and guide other countries in their development, planning and implementation of a national accreditation process for midwifery education.

## Background

High-quality education is critical when it comes to increasing the professional competence of midwives so that they are equipped to provide high standards of safe, evidence-based sexual, reproductive, maternal and newborn care [1–3]. If high-quality midwifery education is to be achieved globally, and in Bangladesh in particular, the implementation of the midwifery education programme needs to be accelerated to ensure that it leads to competency, provides service users with high-quality midwifery care and allows employers’ access to professionals who can perform to international standards and develop towards a level of excellence [4,5]. The 38 public nursing colleges/institutions that offer midwifery education in Bangladesh [6] need to further align their programmes so that the result is more coordinated midwifery education across the country and so that educational bodies and clinical practice sites receive the support they need. Importantly, this will ensure that students are well prepared for their future profession [7].

One way to assure quality and accountability in midwifery education is through the implementation of a credible, comprehensive and effective accreditation system [8]. This is in line with the WHO’s global directives for strengthening nursing and midwifery [9], the Global Strategy on Human Resources for Health: Workforce 2030 [8], and the Midwifery Education Accreditation Programme that was recently developed by the International Confederation of Midwives (ICM) [10]. The aim of accreditation is to ensure that students graduate at an acceptable level so that they can meet the healthcare needs of patients and the wider population. Once accredited by the relevant government agency, educational institutions can then exercise their power both to award degrees and to license and certify graduates for professional practice [11]. Given that fewer than half of all countries worldwide have an accreditation system in place for midwifery education [12,13], it will be difficult for any to argue that they offer quality education unless the educational programmes and institutions are accredited. As such, in 2017, the Bangladesh Government developed an accreditation assessment tool that involved local engagement. This was to make successful and culturally sensitive development and implementation of an accreditation assessment system possible [14].

Bangladesh has made significant progress in developing midwifery education since the initial three-year programme leading to a diploma in midwifery in 2013. It has established partnerships between government, academia, professional bodies, non-governmental organisations and donors [15]. With extensive investment in building the capacity of nursing educators to shift their focus from teaching nursing to teaching midwifery [16], the development of an accreditation tool for midwifery education sites, along with a set of guidelines, was one way to build the capacity of midwifery educators and to enable them to contribute to policy, as well as academic and healthcare development and implementation [17]. In 2016, 113 educators completed a post-basic midwifery programme. This was followed by a master’s programme for midwifery educators [18], which 120 midwifery educators completed in 2019. The awareness of the benefit of quality midwifery education [1–3] has led to the development of a mentorship programme [19], a simulation-based learning programme for midwifery educators [20] and a Massive Open Online Course (MOOC), contextualised for Bangladesh, on the role and function of midwives, which serves to guide midwives at the political, management, academic and practice levels [16].

Factors influencing the implementation of quality midwifery education can be assumed to vary, as can the levels of investment that governments are willing to put towards this initiative. It is therefore important to understand the factors that have facilitated the implementation of an accreditation system in Bangladesh. As such, the aim of this study is to describe both positive and negative aspects of the implementation of an accreditation process at midwifery education institutions in Bangladesh and to sketch out areas for possible improvement.

## Method

### Study design

A qualitative research design was used. This is a useful approach, especially when little is known about a subject [21], in this case the positive and negative aspects related to the implementation of an accreditation process at midwifery education institutions in Bangladesh. Data was collected through focus group discussions (FGD) with policymakers, regulatory authorities and midwifery educators, and was analysed using content analysis with a deductive approach inspired by Elo and Kyngäs [21]. The Consolidated Framework for Implementation Research (CFIR) [22] was used as a theoretical framework to guide the data analysis and description of the results. Ethical clearance was obtained from the Ethical Review Committee: Centre for Injury Prevention and Research, Bangladesh CIPRB/ER/2018/12.

### Setting – Bangladesh, midwifery education and its governing bodies

Bangladesh is a low-middle income country with approximately 3.1 million live births per year [23]. Forty-seven percent of deliveries take place in a health facility, which contributes to an overall low ratio of skilled attendance at birth of 50% and a maternal mortality ratio of around 196 maternal deaths for every 100,000 live births [24].

In Bangladesh, midwifery education has, since its inception, been firmly placed at nursing institutions and colleges. Delivered at a diploma level, the midwifery programme consists of 60% clinical practice and 40% theory, and is currently being delivered at 38 public educational institutions, geographically distributed across the country [6]. A nursing college is a government institution at the divisional level, affiliated with tertiary hospitals with a high level of referrals from the primary level. Nursing institutions are situated at the district level and are affiliated with general hospitals where midwifery students are placed for their practical learning placements. The yearly intake of midwifery students is 25–50 places for each educational institution. These are residential, and students come from both rural and urban areas. This study involves all (n = 38) public educational institutions delivering midwifery diploma education and their collaboration for clinical placements with affiliated hospitals.

The Directorate General of Nursing and Midwifery (DGNM) and the Bangladesh Nursing and Midwifery Council (BNMC) govern nursing and midwifery education and services. The Directorate is responsible for recruiting midwifery students and educators, and for deciding at which educational institution they will study or work. It is also responsible for ensuring high-quality and safe clinical placements for midwifery students and for determining the creation of new midwifery positions and the deployment, promotion, transfer and retention of existing midwives. The Council is an autonomous regulatory body for nursing and midwifery education and services, and falls under the medical education and family welfare division of the Ministry of Health and Family Welfare. The BNMC ensures educational standards, and develops and approves curricula for midwifery education and practice. The Council also validates public and private nursing and midwifery educational institutions and provides for the registration of nurses and midwives, their licensing and its renewal. It was under the guidance of the DGNM and BNMC that the context-specific accreditation assessment tool was developed so as to uphold quality midwifery education, which was piloted in 2017.

### Development of the context-specific accreditation assessment tool

The context-specific accreditation assessment tool was developed using a participatory consensus-building approach. Involved in its development were the Bangladesh Government, national and international academia, the midwifery association, and donor and technical agencies. The aim was to uphold quality midwifery education in Bangladesh and to prepare for the upcoming global Midwifery Education Accreditation Programme [10]. Through the use of the ICM Global Standards for Midwifery Education [25] and the WHO’s Midwifery Educator Core Competencies [26], global level documents were adapted for the Bangladeshi setting. The assessment tool builds on five domains: (1) organisation and administration, (2) midwifery faculty, (3) student body, (4) curriculum and (5) assessment strategies. These domains cover the minimum requirements to achieve quality in midwifery education, institutions and practical learning placements, and is specifically aligned with the learning outcomes of the 3-year midwifery diploma education programme [14]. The assessment tool with its 14 educational standards and 37 multiple-choice closed-response questions in English has been approved by the DGNM and BNMC.

### Data collection and participants

Prior to data collection, a one-day training course was organised for the *Accreditation Committee* selected by the Bangladeshi Government. Ten midwifery educators, who were registered in a master’s programme in Sexual, Reproductive and Perinatal Health Care, attended. They were there to be trained as data collectors. First, the researchers (MB and KE) gave a presentation of the accreditation assessment tool and guidelines for its use, which all committee members had helped to develop. This was followed by an introduction on how to use the interview guide. Accreditation Committee members were able to discuss issues and ask questions after the presentation and introduction. Lastly, participants were divided into five assessment teams. Each team was allocated a set number of educational institutions to visit, as determined by the BNMC. Shortly thereafter, the assessment of the 38 educational institutions took place using the context-specific accreditation tool. The assessment included focus group discussions (FGDs). This study presents only the data collected from the FGDs.

Data was collected from the focus groups immediately after the accreditation assessment had been conducted. Each focus group comprised 1) policymakers and regulatory authorities, 2) the Accreditation Committee and 3) midwifery educators involved in midwifery education, regulation and services. The FGD were held in a separate room at the educational institutions between February and July 2018. In total there were 276 participants (only one was male), who were divided into 40 focus groups, each with 4–9 participants. The average age of each group was 50 years with an average of 25 years of work experience in clinical nursing and midwifery or as a faculty member. One participant had a PhD, 29 had bachelor’s degrees and 21 had diplomas in nursing and midwifery. The remaining 225 participants had master’s degrees in Public Health/Sexual, Reproductive and Perinatal Health or in Nursing and Midwifery. The characteristics of the participants are given in Table 1. The FGDs with educators (n = 38) were held at their respective institutions and were led by the accreditation committee members. The FGDs held at government offices were led by MB (n = 1) and KE (n = 1). At the FGDs, one of the two committee members presented the context-specific accreditation tool and posed the two discussion questions:

- What is your perception of the context-specific accreditation tool for midwifery education that is in use at your institute?
- What are the positive and negative aspects of implementing the context-specific accreditation tool for midwifery education?

**Table 1.** Characteristics of participants (n = 276)

	Female	275
Gender	Male	1
Age range		35–59
Mean		49,33
Designation	Nursing Instructor/Educator	164
	Nursing Instructor in charge	11
	Principal	8
	Senior Staff Nurse	49
	Nursing Supervisor	11
	Nursing Superintendent	12
	Government employee	20
Academic qualification	PhD	1
	Masters’degree in Public Health/Sexual, Reproductive and Perinatal Health/Nursing and Midwifery	226
	Bachelor degree	29
	Diploma in Nursing and Midwifery	21
Number of working experience	<10	12
	11–20	70
	21–30	69
	>31	124

In total there were 276 participants, only one was male, divided into 40 focus groups, each consisting of 4–9 participants. The average age of each group was 50 years with an average working experience in clinical nursing and midwifery or as a faculty member of 25 years. One participant had a PhD, 29 had bachelor’s degrees and 21 had diplomas in nursing and midwifery. The remaining 225 participants had master’s degrees in Public Health/Sexual, Reproductive and Perinatal Health or in Nursing and Midwifery

All participants were given information orally about the study and their rights: one such right was that they could refrain from answering a question or discontinue the interview without explanation at any time [27]. In line with the procedures of the FGDs [28], the committee member who acted as an assistant posed probing questions such as ‘Can you give another example?’ or ‘Do you have one more example?’ and made sure that all participants were included in the discussion. All FGDs were audio recorded and lasted 45–70 minutes.

### Theoretical framework

To describe both positive and negative aspects of the implementation of an accreditation process at midwifery education institutions in Bangladesh, the CFIR framework [22] was applied during data analysis and description of the results. The CFIR is a synthesis of concepts described in 19 implementation frameworks, models and theories, and organises 39 constructs across five domains: 1) intervention characteristics (features of an intervention that might influence implementation), 2) inner settings (features of the implementing organisation that might influence implementation), 3) outer settings (features of the external context or environment that might influence implementation), 4) characteristics of individuals (characteristics of individuals involved in implementation that might influence implementation) and 5) process (includes strategies or tactics that might influence implementation). The framework was chosen as it is flexible in application and well suited to guiding work to change a system so as to improve intervention outcomes; further, the framework has been found to be useful in research areas such as healthcare science, clinical management and nursing [22,29,30]. Table 2 illustrates the application of the five CFIR domains related to the implementation of midwifery education accreditation in Bangladesh.

**Table 2.** Illustration and description of the CFIR domains and sub domains, and its application in this study

Domains of CFIR	Sub Domains of CFIR	Short description	Application in this study
Intervention Characteristics	Intervention source	Participant’s perception about whether the intervention is externally or internally developed.	Participant’s perception about how the assessment tool had been developed
	Evidence, strength and quality	The participant’s perception of the quality and validity of evidence supporting the belief that the intervention will have desired outcomes.	Participant’s perception and knowledge about the accreditation assessment tool
Outer Setting	Needs and resources	The extent to which patient needs, as well as barriers and facilitators to meet those needs, are accurately known and prioritized by the organization.	The need of including staff from the clinical sites
	External policies	Extent to which an organisation has considered policies, regulations, guidelines, research etc.	The link to national and international policies
Inner Setting	Network and communication	The quality of formal and informal communications within an organisation.	Improved communication channels at various levels.
	Implementation climate	Extent to which the method will be supported within the organisation.	Conflicts between midwifery and nursing educators, and conflicts between midwifery and nursing students.
Characteristic of individuals	Knowledge and beliefs about the intervention	Attitudes toward the method, as well as familiarity with facts, truths, and principles.	Strong believe and positive attitude about the accreditation process.
Process	Planning	Extent to which the implementation of the intervention has been planned in advance, with the purpose to make implementation effective.	A complex planning process
	Engaging	Attracting and involving appropriate individuals in the implementation and use of the intervention through a combined strategy of social marketing, education, role modelling, training, and other similar activities	Level of engagement.
	Reflecting and evaluating	Feedback about the progress and quality of implementation followed up with regular personal and team debriefing about progress and experience. The focus here is specifically related to implementation efforts.	Monitoring and follow up plans to improve quality.

### Data analysis

The audio-recorded FGDs were transcribed verbatim from Bengali to English by an interpreter and were analysed by MB and KE using a deductive content analysis approach inspired by Elo & Kyngäs [21]. First, the transcriptions were read several times so that an understanding could be gained of the positive and negative aspects of the implementation of an accreditation process at midwifery education institutions in Bangladesh. Next, all texts (meaning units) relevant to the aim were sorted into the five domains of the CFIR [22]. Thereafter, the meaning units were read again and organised into ten identified relevant sub-domains of the framework.

## Results

In this study, quotations from policymakers and regulatory authorities are labelled as FGD 1. Quotations from the Accreditation Committee are labelled FGD 2, while those FGDs from midwifery educators are labelled FGD 3–40.

### Intervention characteristics

#### Intervention source

The accreditation assessment tool was developed by the Accreditation Committee as a response to the vision of the Prime Minister of Bangladesh to ensure high-quality, evidence-based midwifery education and services in Bangladesh. The tool was developed using a participatory and consensus-building approach under the guidance of the DGNM and BNMC. *Good that it was done bottom-up and good to be part of the government-formed accreditation committee and of each step when developing and implementing the assessment tool* (FGD 2). Other participants reported that the participatory approach had brought about a sense of ownership among actors, such as decision-makers, policymakers, UN agencies and midwifery educators, and had created a foundation for the implementation of a set of agreed accreditation standards.

#### Evidence, strength and quality

Overall, participants were pleased with the content and structure of the tool, and were keen to see it be implemented. They felt that it had been developed according to international standards, and that they were acceptable to midwifery education in Bangladesh and workable within its emerging midwifery profession. They were pleased to see that the content of the tool contributed to the establishment of a functioning administrative system for the midwifery education programme at each educational institution. They also noted, with approval, that the tool assessed the educators’ formal training in midwifery and their ability to teach evidence-based midwifery in both theory and practice.

*It is good to have assessments and guidelines like this. This will enhance the capacity of evidence- based learning and contribute to evidence-based midwifery services. If the educators and students follow these assessment guidelines, we will make fewer mistakes and the education will function more smoothly* (FGD 28).

In general, participants felt the assessment tool was easy to follow. The actual assessment process involved a series of simple yes/no questions. These helped to identify the extent to which midwifery education programmes and the institutions delivering them had achieved the set standards and which areas still required improvement. *This assessment is very good because it allows us to see the gaps, and makes us aware of the different procedures to reach standards* (FGD 29). That said, participants suggested that a space for free-text responses be added so that a reason behind the yes/no alternatives could be given.

### Outer setting

#### Needs and resources

It was noted that the clinical placement sites used by midwifery students were not included in the assessment process and nor were clinic staff aware that the assessment process existed and was being implemented. The importance of including staff from the clinical sites in the assessment process was stressed because an imbalance was noted between the evidence-based practices being taught to new students at the educational sites and the older, largely outdated practices in place at clinics. Unless this divergence is addressed, participants feel it would be impossible for midwifery education programmes and institutions to achieve the accreditation standards.

*The midwifery students know how to delay cord cutting, but the senior staff nurses [at the clinical placement sites] do not agree with them and they pushed the midwifery students to cut the cord immediately. So these people need training at least to understand basic midwifery* (FGD 9).

Another negative aspect was that midwifery students had to compete with medical and nursing students for clinical exposure to childbirth, and antenatal and postnatal cases in order to reach their required number of clinical experiences. They were required to have 40 childbirth experiences as part of their midwifery education: achieving 40 clinical birth experiences was difficult, they stated. Focus group participants felt this to be a significant omission and further felt clinical preceptors to be essential.

*We need midwifery clinical preceptors to assure the required clinical experiences as per the curriculum in the midwifery education* (FGD 30).

### External policies

Participants were grateful for the opportunity that the assessment tool gave them to prepare for the global midwifery education accreditation of the ICM, which they hoped to take in the future. They liked the fact that the accreditation tool was linked to high-level government policies, with its development and construction based on them. According to participants, this gave the tool status and authority within the midwifery education sector.

*The Government has taken the initiative to provide quality care to the people through midwives and we should follow that vision* (FGD 8).

The assessment was seen to be ambitious, even though it did not show institutions how to address the identified weaknesses. There was some uncertainty about the country’s capacity to deal with the assessment’s findings, and there was a feeling that it would take a long time to enable institutions to comply with the required standards.

*The assessment standards are beyond our capacity and they are not realistic because those who have planned this do not have much of an idea about the reality. There is no short, mid- and long-term plan in this assessment. To implement these 14 standards will take a long time, but gradually we will improve the implementation* (FGD 13).

### Inner setting

#### Network and communication

The accreditation assessment process improved communication between the educational institutions, policymakers and regulatory authorities. The process required that they contact each other by e-mail, during on-site visits and in video conferences. As part of the accreditation process, the educators communicated with other institutions as a way to support each other in their aim to reach the set standards and also to compare achievements and come with suggestions for improvement.

Once institutions were notified about the assessment, some midwifery educators started to visit clinical sites more frequently. They were prompted to investigate the extent to which their midwifery students were practising what they had been taught. The assessment process caused these educators to reflect on their lack of communication with clinical sites, and encouraged them to adjust their practices. They came to recognise the importance of good communication between the institution and the clinical site for the achievement of students’ learning outcomes.

*To comply with the accreditation standards, we will need to have better communication with and sources of information for the educational institution and the clinical sites, preferably on a day-to-day basis* (FGD 36).

Some educators, however, felt that the accreditation process was a way for the Government to monitor and potentially target them even if they liked the fact that they had better communication with actors and could better reach out to the clinics.

*We do have a fear of being monitored and we will compare our results with other educational institutions. We believe we have gaps and if these are being identified through an assessment, with support from the authorities, we can hopefully overcome these* (FGD 8).

#### Implementation climate

There was competition within the educational institutions, between the educators teaching in the midwifery department and those teaching in the nursing programmes. This rivalry ended up involving the midwifery and nursing students, and affected a number of areas including accommodation, teaching facilities, and availability and use of teaching and learning equipment. Midwifery faculty found it difficult to find adequate classrooms and struggled to gain access to computers and projectors. Midwifery students were often allocated the poorest accommodation. According to the participants, this competition derived from a culture of holding each other back because of resentment of the new discipline and jealousy over status and attention. This led to conflicts within the work and study environment, with midwifery educators and midwifery students being shown a lack of encouragement and respect. *The midwifery education programme is a burden for us at the institution because we already have nursing* (FGD 5).

### Characteristics of individuals

#### Knowledge and beliefs about the intervention

All participants had a positive attitude towards the accreditation assessment and valued its implementation. Those educators who were members of the Accreditation Committee and who were involved in the development of the accreditation tool were particularly knowledgeable about the tool and how it worked, and were eager to address identified weaknesses.

Participants also stated that their involvement in the development process had enabled them to advocate for the accreditation assessment tool within their departments. It was clear that they had inspired their colleagues to support it as well.

*We did not have any assessment tool to measure quality before. I found this accreditation tool very useful for our institution. Personally, I was involved in the development process of this tool. I think every section of this assessment tool is important* (FGD 2).

The policymakers and regulatory authorities expressed a strong commitment to the accreditation process with a feedback-appeal-response system built into it. They felt it important that participating institutions and programmes receive their assessment results. They therefore intended to distribute recommendation letters to each educational institution, followed by a formal reassessment.

### Process

#### Planning

Participants felt the planning procedures were complex. They pointed out that there were many steps to be considered prior to the assessment visit. The accreditation committee had to provide the DGNM with draft invitation letters for the visit to each educational institute. Once the invitation letter was approved, a government order had to be issued. Under the guidance of the BNMC, the Accreditation Committee informed the institution that a visit had been scheduled through email and letters. Phone calls were then made to confirm the date and time for the assessment. A notable strength of the process was that all these steps were anchored at the policy level. A weakness, meanwhile, was the difficulty reaching head staff in the institutions who could confirm the date and time for the assessment visit.

*The heads of the institutions do not check their email regularly, and it takes several days to deliver the letter to them. The other issue is the connectivity because everyone in the assessment committee should be informed to follow up with phone calls* (FGD 2).

The site visits for the accreditation committee were well-planned. However, the accreditation committee did not always feel that the head staff and educators at the institutions welcomed their visits. That said, the midwifery educators themselves stated that they had been proud to take part in the assessment.

*We feel proud to be part of this assessment; our educators are part of the process. It is worth the time to do this. For us it is a matter of pride because it makes our college a pioneer in developing high-quality midwifery education* (FGD 4).

#### Engaging

The participants liked the comprehensive nature of the accreditation process and the fact it involved everyone. This was seen as an important factor for the successful implementation of the accreditation process. Unless the clinical sites and the midwifery students were also involved at each step of the implementation process, the set accreditation standards could not be realised.

*All levels, from policy, education and clinical, are critical for successful implementation and for best outcome of the standards. We all must be engaged so as to ensure that our students have the opportunities to become well-educated midwives* (FGD 1).

#### Reflecting and evaluating

It was deemed important to allocate sufficient time for the assessment. *The assessment is very important but also time-consuming. So how can we manage? Five teachers to teach 215 students and that is not the only thing* (FGD 5).

The proposal came to develop an action plan for each educational institution and to include follow-up visits to monitor the progress being made to meet the set standards. A reassessment by national midwifery assessors should be performed yearly to maintain the accountability of midwifery educators.

*The assessment committee members together with the BNMC and DGNM should recommend improvement areas for each educational site based on data from the assessment. A strong monitoring system will make the teachers more careful to do their duties because they know that they will be monitored and they will have to be accountable* (FGD 1).

## Discussion

By focussing on the implementation of an accreditation process at midwifery education institutions using the CFIR framework, this study closes a critical knowledge gap. It demonstrates how a rigorous accreditation process is necessary for Bangladesh if it is to accelerate high-quality midwifery education that will ensure the provision of quality midwifery care. By using the CFIR, we have studied both the positive and negative aspects of the implementation of an accreditation process at midwifery education institutions in Bangladesh. We have discussed the main findings using the five CFIR domains (*intervention characteristics, inner settings, outer settings, characteristics of individuals and process*) and have identified areas for possible improvement. These have led to the identification of actionable findings that can be used to adjust accreditation processes in different contexts.

One positive aspect demonstrated by the intervention characteristics was the participatory approach. This means that the context-specific accreditation tool was developed and implemented in a Bangladeshi setting, and figured among the country’s education priorities. Similarities can be drawn with Afghanistan, where a nationally owned accreditation system for ensuring and regulating the quality of midwifery education was successfully established and implemented during a period of intensive expansion of midwifery education in 2007 [31]. The fact that the ICM developed a global Midwifery Education Accreditation Programme (MEAP) in 2018 [10] provides countries with an opportunity to harmonise midwifery education standards globally. According to the CFIR [22], adaptability is described as being the extent to which a process can be adapted to meet local needs without jeopardising its core elements. Now that Bangladesh has introduced its accreditation process, it is important to work to improve it by responding to identified national and global needs so as to assure high-quality midwifery education. The suggestion is thus such that the accreditation process be adapted and the assessment tool [17] and its implementation process be aligned without loss of their essential contextual core [22].

The outer setting encompasses external influences, such as strategies and policies [22]. The uptake of quality mechanisms, such as accreditation, is strongly associated with a country’s political climate and governmental cooperation. In contrast, a lack of leadership from a country’s government combined with a lack of grassroots coordination can result in poor integration, consistency and reciprocity in accreditation [32]. It is therefore encouraging that there are existing strategies and policies in Bangladesh [16] that have supported an implementation process that is in turn appropriate for the Bangladeshi setting [17]. The importance of identifying and engaging stakeholders in establishing standards and setting policies for quality-improvement mechanisms, such as accreditation, has been stressed in a systematic review [32]. Thus, Bangladesh already fulfilled the criteria deemed essential for the implementation of a successful accreditation system given the many years of collaboration and the strong relationships that had been formed between stakeholders such as the Government of Bangladesh, non-government organisations, academia, professional associations, United Nations agencies and donors [15]. The WHO has recognised that accreditation is critical when it comes to assuring high quality and accountability in midwifery education, both clinical and theoretical [7,9]. However, as this study has shown, implementing an accreditation process was seen to be ambitious, and participants were uncertain about the country’s capacity to reach the set standards. It is both encouraging that the United Nations Population Fund (UNFPA) in Bangladesh has supported the development of the accreditation tool and its implementation [17], and reassuring to know that this development has led to the adoption of the WHO’s guidelines on accreditation on nursing and midwifery education in Bangladesh (personal communication UNFPA Bangladesh, 5/9-2019).

Given the fact that 60% of midwifery education in Bangladesh takes place in clinical placements [6], it is striking that healthcare professionals at these sites, seemingly key players in the education of Bangladeshi midwifery students, were not included in the assessment process, and nor were they informed of its existence and implementation. In accordance with implementation science [33], successful implementation in healthcare depends on committed healthcare professionals being part of all steps in the implementation process and on the process being anchored within management so that it can be successfully implemented [4,5]. Ideally, the entire assessment process should include policymakers, regulatory authorities and healthcare professionals representing the clinical practice sites that are involved in delivering the national midwifery curriculum.

The inner setting section explored the implementation process within the educational institutions. One important aspect within this domain was that the accreditation process had made the educators realise that the set standards could not be reached unless these included the extent to which midwifery students were practising midwifery at the clinical sites. Such a measurement, according to the accreditation tool, could be that the midwifery student had experienced at least 40 normal births and had examined 20 full-term newborns [17]. This can be related to findings in an educational article describing how system-wide reforms start with teaching and learning activities and assessment strategies [34]. As the implementation process progressed, the midwifery educators became increasingly aware of the difficulties they faced reaching the set accreditation standards. They then developed new ways of enabling their institutions to reach the standards. This led to a change in their behaviour: they started to visit the clinical sites to make sure their midwifery students actually practised midwifery. The changes brought about by the need to achieve set standards can be expressed as where education leads, improved practice soon follows [35].

Competition between and within the Bangladeshi educational and clinical sites led to a culture of hindering others in their work. The Sustainable Development Goal (SDG) objective is to ensure inclusive and equitable quality education for all (SDG 4) before 2030. Therefore, working together towards a joint goal without competition is necessary in the attainment of SDG 4. In Bangladesh and Nepal, a joint goal was a requirement prior to the launch of the first midwifery education programmes [36].

Our results suggest that competition between nursing and midwifery educators and the failure of midwifery educators to communicate effectively with clinical practitioners will continue to obstruct the implementation of an effective accreditation programme. Unless these areas are addressed, and practice here improved, it will be difficult both to achieve and to sustain high-quality midwifery education in Bangladesh. Strong leadership at the university level has been identified as crucial for the sustainability of midwifery as a discipline within higher education [37]. In this study, communication with leaders at the government level was improved during the accreditation implementation process and can be understood as facilitating the sustainability of midwifery education in Bangladesh.

The behaviour of health workers can influence the implementation of interventions [22]. In this study, the participants stated that in addition to improved communication, involvement by leadership and changes in behaviour, the participation of midwifery educators in the development of the accreditation tool enabled them to advocate for the implementation process within their departments at their respective institutions. It was clear that they had inspired their colleagues to support it as well. This is in line with Grice [38], who stresses involvement and shared practice as two important driving factors for change.

The policymakers and regulatory authorities at the leadership level also expressed a strong commitment to the feedback-appeal-response system that was built into the accreditation process. This was considered the key factor behind change. In conclusion, genuine educational change requires collective effort by all actors at an institution, in particular leaders and educators [38]. This accreditation process and its development can be viewed as one way to drive change. By using the midwifery educators to gather data and by including them in all stages of the implementation process, the midwifery accreditation initiative shows how the capacity of midwifery educators can be increased so they can better utilise the existing academic, healthcare and policy environment [16].

This study found that a real strength of the implementation process was that it had been well anchored at both the policy level and the educational sites. The fact that it was prioritised was seen to be a real benefit, which contributed to the ownership of the process at all levels. As with any implementation process, it is important to closely monitor and review it, and to make necessary improvements [39]. Therefore, organisational leaders need to set out and prioritise their implementation goals and to take into account the likely benefits, costs and resources required, as well as any potential problems and solutions [40]. As such, following the implementation process in Bangladesh, the next steps, as these findings suggest, would be to develop a plan of action to monitor progress towards the set accreditation standards at each of the 38 public educational institutions; to identify the progress made to reach the standards for accreditation; and to estimate the costs and resources needed to achieve the standards. This is in line with implementation science [39,41], which says that any implementation initiative requires close monitoring and calls for changes that are based on identified implementation gaps. In the implementation of a new approach, such as an accreditation process, the steps in moving from the initial assessment to action are to make public the results found at all educational institutions, to encourage acceptance to solicit feedback and suggestions, and to prepare educators, policymakers and donors for future action. Only then will the accreditation process have an impact on the provision of high-quality midwifery education and midwifery care.

### Strengths and limitations

The key strength of this study is that it is the first of its kind to address the implementation of an accreditation process concerning midwifery education in a low-middle income country. Another strength is the use of the CFIR framework [22]. It can be argued that one limitation is that the CFIR was not used to identify determinants that distinguished between high and low implementation success. That said, using the framework made it possible to conduct the analysis without the authors’ preconceived ideas about midwifery education in Bangladesh. Using this framework during an implementation process, as in this study, allows for a critical re-design since it uses the findings to inform adaptation of the accreditation process. Thus, in the next phase, the suggestion is that all educational institutions be reassessed and the results measured in terms of quality improvement towards set standards. The application of CFIR at this stage could then be used to link the determinants of implementation to outcomes, such as implementation and innovation of effectiveness [42]. Two of the authors (AB, SP) and some of the data collectors are government officials responsible for midwifery education and services in Bangladesh. Their presence may, unintentionally, have led some educators to feel controlled and, as a result, reluctant to share negative experiences, despite assurances of confidentiality. However, we trust this potential effect was mitigated by having data collectors who were trained in critical self-reflection about one’s own biases, preferences and preconceptions. The first and last authors (MB and KE, respectively) are two Swedish researchers and midwifery experts, both familiar with the Bangladeshi context. In this study, being a foreigner proved to be a strength in the sense that the participants in FGDs 1 and 2 were keen to explain their experiences during the implementation process to somebody who was not from Bangladesh.

## Conclusion

The description of the positive and negative aspects of the implementation of an accreditation process, along with the findings outlined here, provides insight into a nationally driven accreditation process. This process helped to identify gaps and achievements within midwifery education and practice, and contributed to the WHO’s recommendation that healthcare professionals be accredited. Our findings demonstrate the need for accreditation processes with a built-in feedback-appeal-response system. When a new process such as this accreditation process is being implemented, the steps needed to move from initial assessment to action is to make public the results found at all educational institutions and to encourage acceptance by soliciting feedback and suggestions for future action. Only then can the accreditation process have an impact on the provision of high-quality midwifery education and services.

This paper should encourage other countries to develop, plan and implement a national accreditation process for midwifery education, while reassuring them that the alignment of an accreditation process with international accreditation standards does not mean losing its local contextual character. The process should ideally include midwifery educators, policymakers and regulatory authorities, while also including healthcare professionals representing clinical practice sites involved in clinical midwifery education.

## Data Availability

The datasets used and/or analysed in this study are available from the corresponding author by reasonable request.

## Paper context

In Bangladesh, midwifery education informed by international standards is new and quality, in terms of both theory and practice, remains a challenge. We describe the implementation process of a context-specific accreditation tool used at midwifery education institutions using the CFIR framework. Describing positive and negative aspects of the implementation process has contributed to our understanding of ways to accelerate high-quality midwifery education that will ensure provision of high-quality midwifery care in Bangladesh as well as in similar settings.
